# Conjecturing Harmful Intent and Preemptive Strike in Paranoia

**DOI:** 10.3389/fpsyg.2021.726081

**Published:** 2021-09-08

**Authors:** Yutaka Horita

**Affiliations:** Department of Psychology, Teikyo University, Tokyo, Japan

**Keywords:** paranoia, preemptive strike, aggression, economic games, harmful intent

## Abstract

Paranoia depicts a belief of others having harmful intent. Research using economic games has exhibited the correlation between paranoia and the propensity to characterize ambiguous intentions as harmful. Using a non-clinical sample recruited online from the United States (*N*=290), we examined whether paranoid thoughts influence aggressive behavior against the subjective perception of harmful intent. We conducted a preemptive strike game wherein aggressive behavior was assumed to be guided by the fear of an opponent. The outcomes indicate that (1) individuals with high paranoia assume harmful intent of an opponent more than those with low paranoia (2) conjecturing an opponent’s harmful intent predicted an increase in the probability of a preemptive strike, and (3) paranoia did not have a statistically significant effect on encouraging a preemptive strike. Additionally, the exploratory analysis revealed that paranoia was related to participant’s aggressiveness and with suppositions of other’s self-interests and competitiveness. This study presents empirical evidence that paranoia is related to the perception of social threats in an uncertain situation. We discuss the possibility that paranoid ideation can promote or inhibit a preemptive strike.

## Introduction

Paranoia, a common characteristic of psychosis, is defined as a belief that others have harmful intentions (Freeman and Garety, 2000). Paranoid in the form of persecutory delusion is a prominent symptom of schizophrenia and other psychotic disorders. However, paranoid ideation is a continuum ranging from low- to high-level even among a general population (Freeman et al., 2005; Green et al., 2008; Bebbington et al., 2013; Freeman, 2016; Bell and O’Driscoll, 2018). Therefore, investigating mechanisms of paranoia among non-clinical samples is important not only to understand clinical problems but also paranoia as a general psychological feature.

Paranoia is associated with a tendency to over-attribute negative events to other’s personalistic negative intentions rather than situational factors (Garety and Freeman, 1999; Murphy et al., 2018; Trotta et al., 2021). Studies using hypothetical scenarios, in which the causes of negative events were ambiguous, have shown that people with higher levels of paranoid ideation are likely to rate others’ intentions as hostile than those with lower levels (Combs et al., 2007). These findings indicate that paranoid ideation reflects a cognitive bias that others harbor hostile and malevolent intentions under ambiguous situations: called “sinister attribution error” (Kramer, 1994; Lopes et al., 2018).

Several empirical studies have evaluated the mechanisms of the attribution of harmful intentions in paranoia in genuine social interactions using economic games rather than hypothetical situations (Raihani and Bell, 2017; Saalfeld et al., 2018; Greenburgh et al., 2019; Barnby et al., 2020; Raihani et al., 2021). For example, the Dictator game, wherein a “dictator” decides the amount of money allocated to a “recipient,” is usually conducted to investigate the relationship between paranoid thoughts and attribution of harmful intent. The motivation behind unfair allocations by dictators is ambiguous because dictators might be motivated by self-interest (e.g., desire to maximize payoff) or harmful intent (e.g., desire to reduce recipients’ earnings). Nonetheless, highly paranoid recipients tend to perceive such intentions as more harmful than low-paranoid participants (Raihani and Bell, 2017; Saalfeld et al., 2018; Barnby et al., 2020). Contrariwise, the tendency to attribute intentions of others with self-interest does not differ among recipients with high- and low-paranoid thoughts (Raihani and Bell, 2017, 2018; Saalfeld et al., 2018; Greenburgh et al., 2019; Barnby et al., 2020; Raihani et al., 2021).

Generally, paranoia also predicts reciprocal behavior against the subjective perception of harmful intentions. A questionnaire survey reported that individuals with persecutory delusions develop a propensity toward safety behaviors (i.e., avoiding a perceived threat; Freeman et al., 2007). A meta-analysis of survey data from the general population indicated the association between paranoid ideation and self-reported violence (Coid et al., 2016). Empirical studies using economic games suggest that paranoid thoughts restrict pro-social behaviors. For instance, paranoid thoughts are associated with defection in Prisoner’s Dilemma (Ellett et al., 2013; Savulich et al., 2018), reduce trusting behavior in the Trust game (Fett et al., 2012), and generate low generosity in the Dictator and Ultimatum games (Raihani and Bell, 2018; Savulich et al., 2018). Another study highlights no effect of paranoia on predicting generosity (Raihani et al., 2021). In economic games, paranoia also predicts punitive behaviors or sentiments toward the opponents (Raihani and Bell, 2018). These punitive behaviors are mediated by the attribution of harmful intent (Raihani and Bell, 2018).

In the present study, we observed whether paranoid thoughts affected aggression against harmful intentions using the Preemptive Strike Game (PSG; Simunovic et al., 2013). In PSG, two players are matched, and both receive money as an endowment (e.g., 0.5 dollars). Each player is given an option to attack their opponent, and they decide whether to attack or not within a specific time limit (e.g., 30s). If both decide not to attack their opponent, they receive the initial amount of money without any deductions (i.e., 0.5 dollars). If one player decides to attack their opponent first, the one attacked loses a large sum of money (e.g., the attacked player loses 0.5 dollars). However, the player who attacks their opponent must pay monetary costs, which are smaller than those imposed upon the attacked opponent (e.g., the attacking player loses 0.1 dollars and receives 0.4 dollars).

In PSG, a rational player wishing to maximize payoff will never be involved in a preemptive attack because of the cost associated with the attack. Players believing that their opponent will be encouraged by payoff-maximization also have no reason to attack. Nonetheless, people presuming the irrationality or harmful intent of the opponent may engage in the attack preemptively. Previous studies provide empirical evidence to support this prediction as a substantial proportion of participants chose to attack in the PSG (Simunovic et al., 2013; Mifune et al., 2016, 2017; Halevy, 2017; Jing et al., 2017; Kawada et al., 2019).

Several studies indicate that the attacks in the PSG are primarily triggered by fear rather than spite (i.e., preference for reducing the other’s payoffs even though it is costly). For instance, Simunovic et al. (2013) compared the rate of attack in PSG between a condition wherein both players had an option to attack (i.e., “bilateral condition”) and another wherein only one could attack their opponent unilaterally (i.e., “unilateral condition”). They observed a substantial proportion of attacks in the bilateral condition, while the attacks in the unilateral condition were rarely observed (i.e., only one out of 26 participants attacked). They also conducted an additional hypothetical experiment after completion of the real PSG where participants were asked to imagine that they were given another exit option. If they chose the exit option, both two players lost the same amount of money, which was identical to the cost of selecting the attacking option (e.g., 0.1 dollars), but they could disable the opponent’s attack without receiving a large amount of loss (e.g., 0.5 dollars) by the opponent. Switching from the attack to the exit option means that the attacks in the PSG are based on self-defense rather than harming the opponent. Simunovic et al. (2013) reported that most of the attackers in the real PSG (i.e., 13 of the 16 attackers) preferred the exit option to the attacking one. A subsequent study that conducted the PSG with similar options also found consistent results (Jing et al., 2017). Other studies have shown that subjective perception of threats posed by outgroups (Jing et al., 2017) and the risk of being attacked (Halevy, 2017) facilitate attacks more in the PSG. An interconnection between attacking in the PSG and arginine vasopressin, a hormone in animals known for defensive aggression against intruders, has also been reported (Kawada et al., 2019).

The current study demonstrated the following issues using the PSG experiment. (1) We investigated whether paranoia induces thoughts about others harboring harmful intents in ambiguous social interactions. Previous studies using the Dictator game have asked participants to attribute actions already taken by others and have examined the association between paranoid ideation and the tendency to attribute harmful intentions (Raihani and Bell, 2017; Saalfeld et al., 2018; Barnby et al., 2020). In contrast, we used PSG and asked participants to rate the opponent’s intention before being informed of their action. We examined whether paranoid thoughts induce overestimation of other’s hostility under uncertain situations, in which the actions of others are unknown. (2) We demonstrated whether paranoid thoughts promote aggression against the perceived threat. If paranoid ideation is associated with suppositions about harmful intent, it may also encourage costly aggression as a defense mechanism. Some studies outline a connection between paranoia and low generosity, such as defection in economic games (Ellett et al., 2013; Raihani and Bell, 2018; Savulich et al., 2018). Recent studies have argued that paranoia may reflect an orientation toward self-interest (maximizing own payoffs) rather than distrust (Raihani and Bell, 2018; Raihani et al., 2021). PSG is an experimental situation wherein payoff-maximization cannot be a motive for aggression. Using the PSG among non-clinical samples, we examined whether paranoia impacts social behavior based on fear of harmful intent rather than ungenerous behavior driven by self-interest.

## Materials and Methods

### Participants

Data were collected from April–May 2021, utilizing the Internet. Specifically, participants were recruited via the Amazon Mechanical Turk (MTurk)^1^ using MTurk Toolkit provided by CloudResearch^2^ (Litman et al., 2017). We recruited MTurk workers from the “CloudResearch-Approved Participants” pool provided by CloudResearch. The pool consisted of people who had passed CloudResearch’s attention and engagement measures. In addition, to recruit as many workers as possible while maintaining data quality and avoiding experienced workers (Litman and Robinson, 2020), we restricted the qualifications of MTurk workers based on their history of completing MTurk tasks, called “Human Intelligence Tasks (HIT),” under the condition that the number of approved HITs was up to 5,000 HITs and their HIT approval rate was at least 90%.

The present study comprised two stages. First, we administered a pre-survey to acquire the paranoia score. Subsequently, the participants of the pre-survey were recalled and subjected to the experiments of the PSG. We intended to obtain at least 200 participants to ensure robust analyses and recruited 500 United States residents for the pre-survey, anticipating that the number of participants would decrease in the subsequent PSG. Finally, 290 participants (173 females and 117 males) participated in the PSG experiment. The mean age of participants was 38.41±13.18years (range 18–82years).

### Procedure

#### Pre-survey

Participants finished the Green Paranoid Thoughts Scale (GPTS; Green et al., 2008), which constitutes 32 items to gauge the strength of paranoia, and participants rated each item on a five-point Likert scale (1: not at all and 5: totally). Scores range from 32 to 160, with a higher score denoting a greater degree of paranoid thoughts. We summed the marks of the 32 items for each participant to represent their paranoia score, and the questionnaire forms were developed using Qualtrics.^3^ Afterward, participants were asked about their age and gender. For participation in the pre-survey, each candidate was paid 0.50 United States Dollars (USD). In the Supplementary Materials, we present the descriptions of the pre-survey and each item of the GPTS.

#### Preemptive Strike Game Experiment

For the participants, the PSG was announced seven days after the completion of data collection in the pre-survey, and recruitment was stopped fivedays after the sending of invitations. The web-based interface of the PSG was developed using oTree (Chen et al., 2016).

Initially, participants were instructed about the rules of the PSG. They received 0.50 USD as a reward for showing up to the experiment. Additionally, they could earn a bonus according to the outcome of the PSG. Participants were made aware that they would be paired with another participant in the PSG. Then, 0.50 USD was given as capital, after which they decided whether to press the button displayed on their computer screens within 30s. If neither pressed the button within the allocated time, both received the 0.50 USD as is. If a player presses the button, the one who presses it first loses 0.10 USD from their capital, reducing the amount acquired to 0.40 USD. Conversely, the player who fails to press the button first loses their entire capital and ends up with nothing. Participants played the PSG only once. After the instructions of the PSG, they answered questions to check their comprehension of the PSG. If they did not submit correct answers for all the questions, they could not proceed to the next screen. Following a preparatory 10-s countdown (a countdown timer was not shown on the screen), a decision screen was displayed to the participants containing a button and a 30-s countdown timer.

When either player pressed the button or did not take action within 30s, they advanced to the subsequent screen and responded to post-experiment questions. Participants were presented with five items and rated the extent to which each item matched their thoughts while deciding to press the button or not on a seven-point Likert scale (1: I did not think so at all and 7: I thought so strongly). According to the items used in the previous studies (Raihani and Bell, 2017; Saalfeld et al., 2018; Barnby et al., 2020), we asked participants to rate the degree to which participants predicted whether their opponent’ motives had been based on reducing the participant’s money (harmful intent supposition: “My partner must be planning to reduce the money I get”) and earning lots of money (self-interest supposition: “My partner must be planning to earn lots of money”). For exploratory analysis, we added three items to measure possible thoughts that could logically affect behavior in PSG. Participants also rated the degree to which they predicted whether their opponent’s motives had been based on making a difference in earnings (competitive intent supposition: “My partner must be planning to get more money than me”). They rated the degree that their opponents had anticipated an attack from the participants (prediction of the opponent’s fear: “My partner must be afraid that I will press the button first”). Besides, participants evaluated the degree to which they were motivated to reduce their opponent’s earnings (aggressiveness: “I want to reduce the amount of money my partner gets”). The experiment was concluded after the responses were submitted.

Participants were told that a bonus would be paid after data collection. They were randomly paired with another participant afterward, which determined the bonus each candidate received. The instructions concerning the PSG are provided in the Supplementary Materials.

### Ethics Statement

The present study was approved by the Ethics Board of the Department of Psychology at Teikyo University. Before the beginning of the study, participants were provided a consent form. Retrieval of a completed form from all participants was considered as approval to take part in the study. Participants were informed that their participation was voluntary, and they could withdraw their consent while participating in the study by closing the web page.

### Hypotheses

We tested the following pre-registered hypotheses:^4^

*Hypothesis 1*: Highly paranoid people are more likely to assume the intent of their opponents as harmful than low-paranoid people.

*Hypothesis 2*: People who presume that their opponents will have harmful intent shall choose to attack in the PSG more often than those who do not.

*Hypothesis 3*: Highly paranoid people will decide to attack their opponents more often in the PSG than low-paranoid people.

## Results

The average time to complete the pre-survey and the PSG experiment was 3.87min (*SD*=2.12; the range was 0.83–18.15) and 4.98min (*SD*=2.60; the range was 1.50–19.85), respectively. The results did not change much even when we excluded participants who completed the pre-survey or the PSG quickly (completion time under the bottom 10% quantile). Therefore, we reported the results using all obtained data.

All analyses were conducted using R version 3.6.3 (R Core Team, 2020). The “clm” function in the “ordinal” package was used to perform analyses with the ordinal logistic regression model (Christensen, 2019), and the “ggplot2” package was employed for visualization (Wickham, 2016).

### Paranoia Scale

The reliability of the GPTS was quite high (Chronbach’s alpha=0.98), and the mean score of the GPTS was 54.50 (*SD*=26.55; the range was 32–160). At a similar level (~8%) as reported in the previous studies using MTurk (Raihani and Bell, 2018; Saalfeld et al., 2018; Greenburgh et al., 2019; Raihani et al., 2021), we found 23 persons (7.93%) whose scores were above the clinical mean score of 101.9 reported in the original Green et al. (2008).

### Attack in the PSG

Altogether, 56 out of 290 participants (19.31%) chose to attack their opponent. Among the attackers, the average time to decide to attack was 9.25s (*SD*=7.77; the range was 1.32–29.95).

### Post-experiment Questions

Supplementary Table S1 illustrates the descriptive statistics (i.e., mean, median, standard deviations, and 95% confidence intervals) for each score of the five post-experiment items. Supplementary Table S2 shows the correlation coefficients between these items and the paranoia score, while Supplementary Figures S1 and S2 depict the distributions of each score according to paranoia levels and decisions made in the PSG, respectively.

### Testing the Hypotheses

Participants’ gender (0 = females and 1 = males) and standardized age were used as control variables for all analyses. The standardized scores of each item were utilized and considered as continuous variables when items of the post-questionnaire were added as predictors in regression models. Thus, we assumed that the scores had linear effects on change in the response variable.

#### Hypothesis 1: Highly Paranoid People Are More Likely to Assume the Intent of Their Opponents as Harmful Than Low-paranoid People

**Figure 1:**
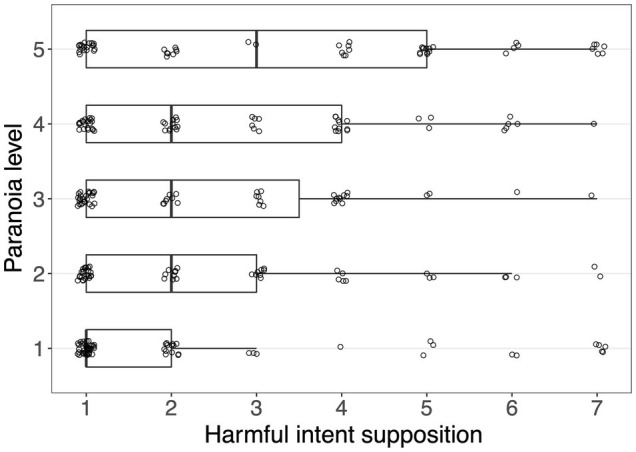
Distributions of presumptions about the opponent’s harmful intent according to paranoia levels. For visualization, paranoia scores were categorized into five levels in terms of quantiles (1, 32–34; 2, 35–39; 3, 40–50; 4, 51–72; and 5, 73–160). Each point represents each participant. Boxplots indicate the distributions of the paranoia score. The box, the thick line in each box, and the whisker represent the interquartile range (IQR), the median, and the distances 1.5×IQR, respectively. Random jitter was added to each point for ease of visibility.

Figure 1 illustrates the distribution of the harmful intent supposition score according to paranoia levels. Table 1 shows the findings of the ordinal logistic regression using the harmful intent supposition score, which was inquired in the post-experiment questions, as a response variable and the paranoia score as a predictor. The result indicated that a higher paranoia score was associated with an increase in conjecturing an opponent’s harmful intent (odds ratio=1.57, 95% CI [1.24, 1.98], *p*<0.001). Hence, hypothesis 1 was supported.

**Table 1:** Results of an ordinal logistic regression model for predicting supposition regarding opponent’s harmful intent.

Parameters	Estimates	[95% CI]	Odds Ratio	[95% CI]	Value of *p*
Intercept 1|2	−0.37	[−0.67, −0.07]			0.014
Intercept 2|3	0.35	[0.05, 0.65]			0.022
Intercept 3|4	0.77	[0.46, 1.08]			<0.001
Intercept 4|5	1.47	[1.12, 1.82]			<0.001
Intercept 5|6	2.14	[1.72, 2.56]			<0.001
Intercept 6|7	2.90	[2.35, 3.45]			<0.001
Paranoia	0.45	[0.21, 0.68]	1.57	[1.24, 1.98]	<0.001
Gender	−0.24	[−0.67, 0.19]	0.79	[0.51, 1.21]	0.271
Age	0.28	[0.06, 0.50]	1.32	[1.06, 1.64]	0.014

*Paranoia score was added as a predictor. Age and gender (0 = female and 1 = male) were used for control variables. All continuous variables were standardized, and the odds ratios were reported for each predictor. CI, confidence intervals*.

#### Hypothesis 2: People Who Presume That Their Opponents Will Have Harmful Intent Shall Choose to Attack in the PSG More Often Than Those Who Do Not

**Figure 2:**
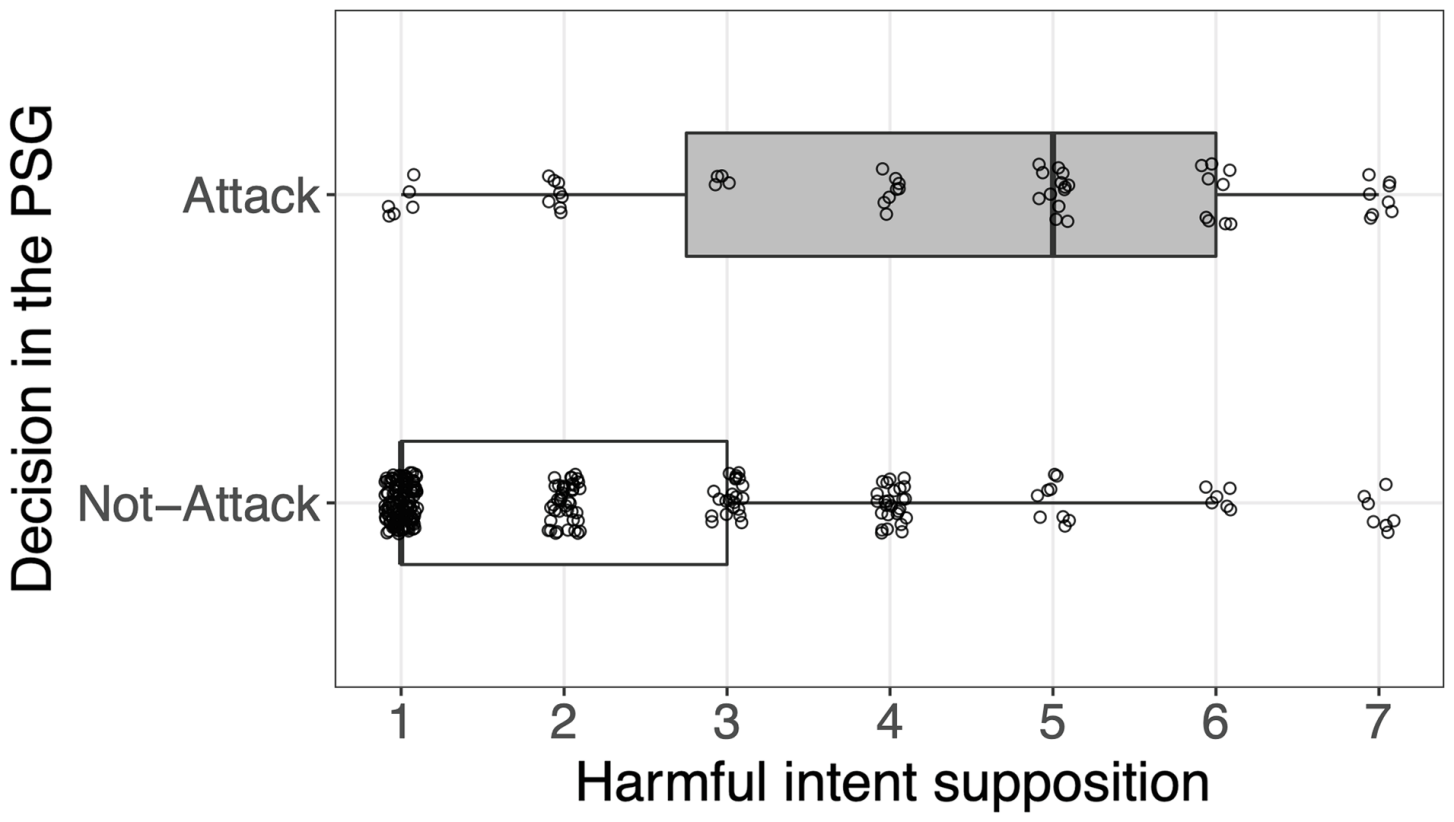
Distributions of supposition concerning the opponent’s harmful intent by decisions made in the PSG. Each point represents each participant. Boxplots indicate the distributions of the paranoia score. The box, the thick line in each box, and the whisker represent the IQR, the median, and the distances 1.5×IQR, respectively. Random vertical jitter was added to each point for ease of visibility.

Figure 2 shows the separate distribution of the harmful intent supposition score according to the decision made in the PSG. Logistic regression was administered by taking the decision made in the PSG (0 = do not attack and 1 = attack) as a response variable and score of the supposition about harmful intent as a predictor. The result indicated that participants who presumed that their counterpart had harmful intent were more willing to attack than those who speculated less (Model 1 in Table 2: odds ratio=1.79, 95% CI [1.51, 2.14], *p*<0.001). Hence, hypothesis 2 was supported.

**Table 2:** Results of logistic regression models that predicted the probability of a preemptive strike.

Parameters	Estimates	[95% CI]	Odds Ratio	[95% CI]	Value of *p*
**Model 1**					
Intercept	−3.36	[−4.19, −2.63]			<0.001
Harmful intent	0.58	[0.41, 0.76]	1.79	[1.51, 2.14]	<0.001
Gender	0.18	[−0.52, 0.86]	1.19	[0.60, 2.37]	0.614
Age	0.38	[0.07, 0.69]	1.46	[1.07, 2.00]	0.018
**Model 2**					
Intercept	−1.50	[−1.92, −1.12]			<0.001
Paranoia	0.29	[−0.02, 0.59]	1.34	[0.98, 1.80]	0.060
Gender	−0.02	[−0.64, 0.59]	0.98	[0.53, 1.80]	0.956
Age	0.53	[0.23, 0.84]	1.70	[1.26, 2.32]	0.001

*The decision in the preemptive strike game (PSG) is employed as a response variable (0 = do not attack and 1 = attack). In Model 1, supposition about the opponent’s harmful intent was added as a predictor. In Model 2, the paranoia score was added as a predictor. In all models, age and gender (0 = female and 1 = male) were utilized for control variables. Supposition about the opponent’s harmful intent was used as a continuous variable. All continuous variables were standardized. The odds ratios were also reported for each predictor. CI: confidence intervals*.

#### Hypothesis 3: Highly Paranoid People Will Decide to Attack Their Opponents More Often in the PSG Than Low-paranoid People

**Figure 3:**
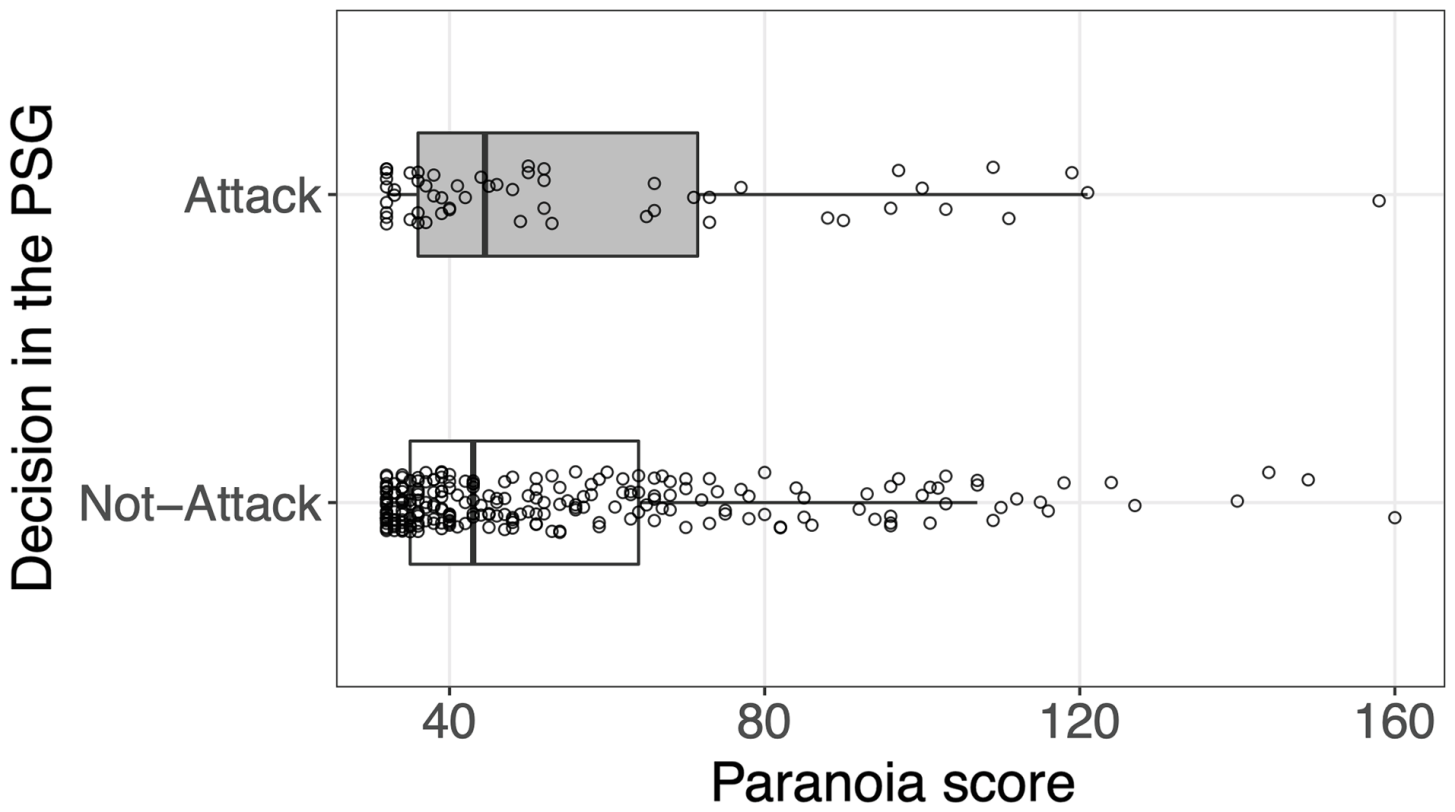
Distributions of the paranoia score in relation to the decision made in the PSG. Each point represents each participant. Boxplots indicate the distributions of the paranoia score. The box, the thick line in each box, and the whisker represent the IQR, the median, and the distances 1.5×IQR, respectively. Random vertical jitter was added to each point for ease of visibility.

Figure 3 shows the distribution of paranoia scores separately according to the decisions made in the PSG. We performed the logistic regression model employing the decision as a response variable and the paranoia score as a predictor. The findings confirm that the effect of paranoia on increasing the probability of attacking in the PSG was marginal and insignificant (Model 2 in Table 2: odds ratio=1.34, 95% CI [0.98, 1.80], *p*=0.060). Hence, Hypothesis 3 was not supported.

We also pre-registered “Hypothesis 3-2”: The relationship between attacking in the PSG and paranoia score will be mediated by conjecturing about the opponent’s harmful intent.^5^ However, Hypothesis 3-2 should only be tested based on the assumption that a significant association between attacking in the PSG and paranoia exists. Since Hypothesis 3 was not supported, we did not test Hypothesis 3-2.

### Unplanned Exploratory Analysis

#### Association Between Supposition Concerning the Opponent’s Self-Interest and Paranoia

Past studies (Raihani and Bell, 2017, 2018; Saalfeld et al., 2018 Greenburgh et al., 2019; Barnby et al., 2020) declare that paranoid thoughts attribute harmful intent rather than self-interest to the counterpart. Supplementary Table S3 shows the results of ordinal logistic regression models using the supposition of self-interest as a response variable. In contrast to previous research, we discovered that the paranoia score predicted more the supposition of other’s self-interest (Supplementary Table S3; odds ratio=1.34, 95% CI [1.08, 1.68], *p*=0.009). We also conducted a logistic regression model wherein the decision in the PSG was taken as a response variable, including harmful intent, self-interest, and paranoia scores as predictors. Supplementary Table S7 summarizes the findings and indicated that harmful intent conjecturing still had a significant positive effect on increasing the probability of attack in the PSG (odds ratio=3.24, 95% CI [2.22, 4.90], *p*<0.001), whereas speculations regarding self-interest did not (odds ratio=0.83, 95% CI [0.54, 1.24], *p*=0.372). The outcomes emphasize that supposition about the opponents’ harmful intent instead of self-interest affected the preemptive strike.

#### The Association Between Other Post-experiment Inquiries and Paranoia

Supplementary Tables S4–S6 show the results of ordinal logistic regression models using three additional items for exploratory analysis: conjecture about the opponent’s competitive intent, prediction of the opponent’s fear, and participants’ aggressiveness as response variables, respectively. Paranoia score was positively associated with the supposition of competitive intent (Supplementary Table S4; odds ratio=1.50, 95% CI [1.20, 1.88], *p*<0.001) and participant’s aggressiveness toward their partner (Supplementary Table S6; odds ratio=1.56, 95% CI [1.22, 2.00], *p*<0.001). Moreover, the influence of paranoia on predicting opponent’s fear was marginally significant (Supplementary Table S5; odds ratio=1.23, 95% CI [0.98, 1.54], *p*=0.069).

#### Factors Affecting the Preemptive Strike

In this section, an unplanned exploratory analysis is reported to investigate the best model for predicting the probability of an attack in the PSG. We administered a logistic regression model that obtained all variables, including other items of the post-experiment questionnaire (e.g., self-interest supposition, competitive intent supposition, predicting opponent’s fear, and aggressiveness) used as predictors. We prospected the best model to explain our data using the multi-model averaging method (Burnham and Anderson, 2002). The details of this method are described in the Supplementary Method.

Supplementary Table S8 shows the results of the logistic regression, derived from multi-model averaging. We could not find significant effects of paranoia (odds ratio=1.02, 95% CI [0.86, 1.19], *p*=0.855) and harmful intent supposition (odds ratio=1.09, 95% CI [0.74, 1.61], *p*=0.660) when controlling for scores of other questions. As a whole, competitive intent supposition (odds ratio=3.14, 95% CI [1.81, 5.47], *p*<0.001) and aggressiveness (odds ratio=1.96, 95% CI [1.36, 2.83], *p*<0.001) had a positive significant effect on increasing the probability of a preemptive strike. Furthermore, the supposition regarding the opponent’s self-interest had a significant effect on reducing the probability of a preemptive strike (odds ratio=0.56, 95% CI [0.34, 0.94], *p*=0.028).

## Discussion

The experiment using the PSG revealed that paranoid thoughts were associated with conjecturing harmful intent. In contrast to previous studies using the Dictator games in which the counterpart’s behavior was revealed (Raihani and Bell, 2017; Saalfeld et al., 2018; Barnby et al., 2020), we used the PSG and asked participants to rate the opponent’s intention before being informed of their action. The results indicated that highly paranoid people think about others having harmful intentions even when their behavior is uncertain. Participants who engaged in preemptive strikes presumed that the intentions of their opponents were harmful.

The aim of the current study was to investigate the effect of paranoia on defensive aggression in genuine social interactions using a controlled economic game. However, the effect of paranoia on increasing the probability of preemptive strikes was weak and statistically insignificant. Some models concerning paranoia would suggest interpretations for the weak effect of paranoid ideation on the preemptive strike. The process by which paranoia leads to attack against perceived harm can be complicated and confounded by other situational or personal factors.

Paranoia has been considered an evolutionary adaptive response to social threats (Gilbert, 2001). The use of not only aggressive but also submissive behaviors can be an adaptive strategy to avoid threats. Some previous questionnaire surveys have indicated a link between paranoia and submission toward others (Freeman et al., 2005; Gilbert et al., 2005; Lopes and Pinto-Gouveia, 2013). Depending on the differences in status between self and others, submissive behaviors rather than aggressive ones can be a better defensive strategy against hostility: It would be better for subordinates to submit to dominant others than challenge them (Gilbert, 2001; Lopes and Pinto-Gouveia, 2013). In future research, experimental demonstrations that control for the subjective perception of social rank (e.g., Saalfeld et al., 2018) would provide insights into understanding the role of paranoia on defensive aggression.

Differences in attribution styles of paranoia may also play a role in the occurrence of a preemptive strike. Trower and Chadwick (1995) theorized two types of paranoia: “Poor-me” and “bad-me” paranoia. People with poor-me paranoia believe that they do not deserve persecution and blame others, whereas those with bad-me paranoia believe that they deserve punishment and blame themselves. Individuals with poor-me paranoia may believe that they do not deserve to be attacked and carry out the preemptive strike. In contrast, those with bad-me paranoia may believe that they deserve to be attacked and suppress attacking the opponent. Although paranoia ideation reflects a tendency to overestimate the harmful intentions of others, the occurrence of defensive aggression against it may depend on the subtypes of paranoia. Future research should examine the association between paranoid ideation and fear-based aggression, controlling for paranoia types (i.e., poor-me and bad-me). Other psychological characteristics related to these subtypes, such as depression or self-esteem (Melo et al., 2006; Udachina et al., 2012), should be considered for controlling variables.

We could also consider the reason we could not detect a substantial effect of paranoia on a preemptive strike because of the relatively smaller baseline of attacking in comparison with those outlined in the previous studies (e.g., Simunovic et al., 2013; Mifune et al., 2016; Halevy, 2017; Jing et al., 2017). However, the rate of attack in this study was similar to those reported in several works (e.g., Mifune et al., 2017; Kawada et al., 2019). The diversity of experimental settings in these studies, such as stake sizes, experimental interface (i.e., laboratory or online), samples (i.e., university students or MTurk), sample sizes, and experimental manipulations, may have generated differences in the attack rate. However, extrapolating clear conclusions is not yet possible due to the limited research regarding the PSG. Considering the association between paranoia and behaviors in economic games, results that repudiate past findings have also been reported, perhaps due to experimental settings (e.g., Raihani and Bell, 2018; Raihani et al., 2021). Hopefully, future research will accumulate empirical evidence on the robustness of the impact of paranoia on social behaviors.

The unplanned exploratory analysis highlighted some limitations of the present study. Contrary to previous findings (Raihani and Bell, 2017, 2018; Saalfeld et al., 2018; Greenburgh et al., 2019; Barnby et al., 2020), the paranoia score predicted the supposition about other’s self-interest. Although the attack in the PSG is never based on maximizing the payoff, the attack may be interpreted as an attempt to earn the least amount of money, even if it is costly. Therefore, paranoid players might think that their opponents would be driven by monetary gains. The lack of measurements that distinguish between payoff-maximization and payoff-optimization remained a limitation of this study. In a sense, the result may reflect negative evaluations of others in paranoia (e.g., Fowler et al., 2006): Paranoid individuals are likely to make negatively biased judgments that the others have selfish orientations (i.e., caring about one’s own interests at the expense of other’s interests). However, when controlling for other factors, conjecturing other’s self-interest no longer had a positive influence on the increasing probability of a preemptive strike. Instead, overall, the presumption concerning other’s self-interest decreased its occurrence. We found that the supposition of other’s self-interest did not have a significant role in determining the incidence of a preemptive strike.

Other exploratory analyses showed that the supposition of competitive intentions was related to a higher level of paranoia. Besides, the best model fit to our data specified that the supposition of competitiveness predicted the probability of a preemptive strike rather than a presumption regarding harmful intent. “Competitiveness” here represents the motivation to increase the difference in payoffs (Messick and McClintock, 1968; Van Lange et al., 1997). Considering the payoff structure of the PSG, we can speculate that competitive intents measure the same concept as concerns about the other’s attack (i.e., harmful intent). Conjecturing competitive intent positively and strongly correlated with that of harmful intent (Supplementary Table S2; *r*=0.79, *p*<0.001). However, a belief that the opponent has competitive motives is irrational because a choice to attack is costly for them. This finding also supports the argument that paranoia reflects an irrational interpretation bias that others have harmful intentions (Kramer, 1994; Garety and Freeman, 1999; Combs et al., 2007; Lopes et al., 2018; Murphy et al., 2018; Trotta et al., 2021). The weak and insignificant association between paranoia and prediction of other’s fear may reflect an inclination of people with paranoia to pay more attention to the hostility of others than to their anxiety.

Participant’s aggressiveness was also positively associated with their paranoia score. Past research mentions that paranoid ideation diminishes pro-sociality (Savulich et al., 2018; Raihani et al., 2021) and correlates with the self-reported experience of reward through negative social interactions (Raihani et al., 2021). These results may be consistent with our findings, wherein people with highly paranoid thoughts self-reported that they wanted to reduce their opponent’s payoffs. Exploratory analysis using all obtained variables revealed that overall participant’s aggressiveness also significantly predicted an increase in the probability of a preemptive strike.

However, based on various evidence, we should doubt whether offensive motivation has a pivotal role in encouraging an attack in the PSG. First, the participants in this study had scored relatively low on the item of aggressiveness, even among those who had decided to attack (Supplementary Table S1; the median was three on a seven-point rating). Second, as a limitation of the present study, self-reports after decision-making seemed unreliable to measure the motivation behind actions. Participants might retroactively specify the reason behind their action as the action they had already performed. Third, as described in the introduction, previous studies have consistently provided empirical evidence that attacking in the PSG occurs as fear-based defensive aggression rather than an offensive one (Simunovic et al., 2013; Halevy, 2017; Kawada et al., 2019). However, we cannot logically deny that the motivation to launch an offensive could work in the PSG, where both players have an option to attack each other. Notably, intimidation is speculated to inflame more competition. Future research is necessary to control for pre-measured participants’ pro-sociality (e.g., Messick and McClintock, 1968; Van Lange et al., 1997) or aggressiveness (e.g., Buss and Perry, 1992) to evaluate the motivation behind preemptive strikes and its relationship to paranoia.

Cognitions or behaviors related to coalitions, such as detection or avoidance of social threats, are considered the evolutionary foundation of paranoia (Gilbert, 2001; Green and Phillips, 2004; Raihani and Bell, 2019; Bell et al., 2021). From this perspective, recent works have investigated the role of paranoia in detecting social threats using experiments wherein group affiliation, relative social status, and group cohesion are manipulated (Saalfeld et al., 2018; Greenburgh et al., 2019). These previous studies debate that paranoia represents a lower threshold for fear of social threats. The present study also proposes evidence that paranoia functions as detecting social threats. There is room for the present study to be enhanced; for instance, the PSG should be implemented in intergroup or individual vs. group conflict situations (Mifune et al., 2016; Jing et al., 2017; Kawada et al., 2019). Further experimental studies with non-clinical groups would bolster understanding about normal psychological mechanisms of paranoia as an adaptive response to external threats.

## Data Availability Statement

The datasets presented in this study can be found in online repositories. The names of the repository/repositories and accession number(s) can be found at: Open Science Framework (link: https://osf.io/9vqfc/).

## Ethics Statement

The studies involving human participants were reviewed and approved by Ethics Board of the Department of Psychology at Teikyo University. The patients/participants provided their written informed consent to participate in this study.

## Author Contributions

The author confirms being the sole contributor of this work and has approved it for publication.

## Funding

This study was financially supported by the JSPS (Japan Society for the Promotion of Science) KAKENHI (Grants-in-Aid for Scientific Research; grant no. JP18K13276).

## Conflict of Interest

The author declares that the research was conducted in the absence of any commercial or financial relationships that could be constructed as a potential conflict of interest.

## Publisher’s Note

All claims expressed in this article are solely those of the authors and do not necessarily represent those of their affiliated organizations, or those of the publisher, the editors and the reviewers. Any product that may be evaluated in this article, or claim that may be made by its manufacturer, is not guaranteed or endorsed by the publisher.

## Footnotes

^1^ www.mtruk.com

^2^ www.cloudresearch.com

^3^ www.qualtrics.com

^4^ https://osf.io/y97n3/

^5^ https://osf.io/y97n3/
