# Supplementary material for: Conjecturing Harmful Intent and Preemptive Strike in Paranoia
Source: Front Psychol. 2021 Sep 8;12:726081. doi: 10.3389/fpsyg.2021.726081 (PMC8455818; doi:10.3389/fpsyg.2021.726081)
Supplement: Supplementary file 1 [file Data_Sheet_1.PDF]

Supplementary Information for "Conjecturing harmful intent and preemptive strike in paranoia."

Yutaka Horita[1*]
1. Department of Psychology, Teikyo University, Tokyo, Japan

Supplementary Tables

Table S1: Descriptive statistics for each score of post-experimental items.
Table S2: Correlation coefficients between each measure.
Table S3: Results of an ordinal logistic regression model with the conjecture concerning the opponent's self-interest as the response variable.
Table S4: Results of an ordinal logistic regression model with the conjecture concerning the opponent's competitive intent as the response variable.
Table S5: Results of an ordinal logistic regression model with the prediction of the opponent's fear as the response variable.
Table S6: Results of an ordinal logistic regression model with the participant's aggressiveness as a response variable.
Table S7: Results of logistic regression models with the decision made in the PSG as the response variable controlling for assumptions regarding both the opponent's harmful intent and self-interest.
Table S8: Results of the best model to predict the probability of an attack in the PSG.

Supplementary Figures

Figure S1: Distributions of post-experimental questionnaire items by paranoia levels.
Figure S2: Distributions of each score of post-experimental questionnaire items in relation to decisions in the PSG.

Supplementary Method

Model selection with model averaging

Supplementary Materials

Instructions for the pre-survey to assess the Green et al. (2008)'s Paranoid Thoughts

Scale.

Instructions for the preemptive strike game.

Supplementary Table S1: Descriptive statistics for each score of post-experimental items. The table reported the statistics calculated by pooling decisions in the PSG and those calculated separately by decisions in the PSG. The number inside parentheses indicates standard deviations (SD). CI: confidence intervals.

	Mean (SD)	Median	95% CI
Self-interest	3.10 (2.14)	2	[2.86, 3.35]
Not-Attack	2.94 (2.12)	2	[2.66, 3.21]
Attack	3.80 (2.10)	4	[3.25, 4.35]
Harmful intent	2.58 (1.87)	2	[2.36, 2.79]
Not-Attack	2.16 (1.60)	1	[1.96, 2.37]
Attack	4.30 (1.93)	5	[3.80, 4.81]
Competitive intent	2.88 (2.07)	2	[2.64, 3.12]
Not-Attack	2.43 (1.79)	2	[2.20, 2.66]
Attack	4.77 (2.09)	5	[4.22, 5.31]
Prediction of fear	4.16 (2.02)	4	[3.92, 4.39]
Not-Attack	4.04 (1.99)	4	[3.78, 4.29]
Attack	4.64 (2.11)	5	[4.09, 5.20]
Aggressiveness	1.84 (1.61)	1	[1.66, 2.03]
Not-Attack	1.49 (1.21)	1	[1.33, 1.64]
Attack	3.32 (2.15)	3	[2.76, 3.88]

Supplementary Table S2: Correlation coefficients between each measure. * $p < 0.05$, ** $p < 0.01$, *** $p < 0.001$.

	1	2	3	4	5	6
1. Paranoia		0.17**	0.19**	0.16**	0.11	0.13
2. Self-interest			0.48***	0.56***	0.45***	0.38***
3. Harmful intent				0.79***	0.34***	0.63***
4. Competitive intent					0.43***	0.54***
5. Prediction of fear						0.29***
6. Aggressiveness						

Supplementary Table S3: Results of an ordinal logistic regression model with the conjecture concerning the opponent's self-interest as the response variable. Age and gender (0 = female, 1 = male) were used for control variables. All continuous variables were standardized. The odds ratios were also reported for each predictor. CI: confidence intervals.

Parameters	Estimates	[95% CI]	Odds Ratio	[95% CI]	*P* Value
Self-interest					
Intercept 1|2	–0.32	[–0.61, –0.03]			0.029
Intercept 2|3	0.17	[–0.12, 0.46]			0.258
Intercept 3|4	0.50	[0.20, 0.79]			0.001
Intercept 4|5	1.04	[0.72, 1.36]			< 0.001
Intercept 5|6	1.74	[1.38, 2.10]			< 0.001
Intercept 6|7	2.38	[1.94, 2.81]			< 0.001
Paranoia	0.30	[0.07, 0.52]	1.34	[1.08, 1.68]	0.009
Gender	0.39	[–0.03, 0.81]	1.48	[0.97, 2.26]	0.069
Age	–0.07	[–0.29, 0.15]	0.93	[0.75, 1.16]	0.543

Supplementary Table S4: Results of an ordinal logistic regression model with the conjecture concerning the opponent's competitive intent as the response variable. Age and gender (0 = female, 1 = male) were used for control variables. All continuous variables were standardized. The odds ratios were also reported for each predictor. CI: confidence intervals.

Parameters	Estimates	[95% CI]	Odds Ratio	[95% CI]	*P* Value
Competitive intent					
Intercept 1|2	–0.30	[–0.59, –0.004]			0.047
Intercept 2|3	0.19	[–0.10, 0.48]			0.207
Intercept 3|4	0.60	[0.30, 0.90]			< 0.001
Intercept 4|5	1.21	[0.88, 1.54]			< 0.001
Intercept 5|6	1.75	[1.38, 2.12]			< 0.001
Intercept 6|7	2.50	[2.04, 2.96]			< 0.001
Paranoia	0.41	[0.18, 0.63]	1.50	[1.20, 1.88]	< 0.001
Gender	0.07	[–0.36, 0.50]	1.07	[0.70, 1.64]	0.751
Age	0.28	[0.06, 0.50]	1.32	[1.06, 1.65]	0.014

Supplementary Table S5: Results of an ordinal logistic regression model with the prediction of the opponent's fear as the response variable. Age and gender (0 = female, 1 = male) were used for control variables. All continuous variables were standardized. The odds ratios were also reported for each predictor. CI: confidence intervals.

Parameters	Estimates	[95% CI]	Odds Ratio	[95% CI]	*P* Value
Prediction of fear					
Intercept 1|2	−1.58	[−1.93, −1.23]			< 0.001
Intercept 2|3	−1.10	[−1.41, −0.78]			< 0.001
Intercept 3|4	−0.60	[−0.89, −0.30]			< 0.001
Intercept 4|5	0.09	[−0.19, 0.38]			0.524
Intercept 5|6	0.82	[0.52, 1.12]			< 0.001
Intercept 6|7	1.81	[1.44, 2.18]			< 0.001
Paranoia	0.21	[−0.02, 0.43]	1.23	[0.98, 1.54]	0.069
Gender	0.03	[−0.38, 0.44]	1.03	[0.69, 1.56]	0.875
Age	−0.08	[−0.29, 0.13]	0.93	[0.75, 1.14]	0.475

Supplementary Table S6: Results of an ordinal logistic regression model with the participant's aggressiveness as a response variable. Age and gender (0 = female, 1 = male) were used for control variables. All continuous variables were standardized. The odds ratios were also reported for each predictor. CI: confidence intervals.

Parameters	Estimates	[95% CI]	Odds Ratio	[95% CI]	*P* Value
Aggressiveness					
Intercept 1|2	1.03	[0.68, 1.38]			< 0.001
Intercept 2|3	1.58	[1.20, 1.97]			< 0.001
Intercept 3|4	1.90	[1.49, 2.31]			< 0.001
Intercept 4|5	2.54	[2.06, 3.02]			< 0.001
Intercept 5|6	2.94	[2.40, 3.49]			< 0.001
Intercept 6|7	3.47	[2.81, 4.13]			< 0.001
Paranoia	0.44	[0.20, 0.69]	1.56	[1.22, 2.00]	< 0.001
Gender	0.28	[−0.22, 0.79]	1.32	[0.80, 2.19]	0.276
Age	0.33	[0.07, 0.59]	1.40	[1.08, 1.81]	0.012

Supplementary Table S7: Results of logistic regression models with the decision made in the PSG as the response variable controlling for assumptions regarding both the opponent's harmful intent and self-interest. Age and gender were used for control variables. Both the harmful intent supposition score and the self-interest supposition score were used as continuous variables. All continuous variables were standardized. The odds ratios were also reported for each predictor. CI: confidence intervals.

Parameters	Estimates	[95% CI]	Odds Ratio	[95% CI]	*P* Value
Intercept	−2.96	[−4.15, −1.85]			< 0.001
Paranoia	0.01	[−0.36, 0.36]	1.01	[0.70, 1.44]	0.954
Harmful intent	1.18	[0.80, 1.59]	3.24	[2.22, 4.90]	< 0.001
Self-interest	−0.19	[−0.61, 0.21]	0.83	[0.54, 1.24]	0.372
Gender	0.20	[−0.50, 0.90]	1.23	[0.61, 2.46]	0.566
Age	0.03	[0.003, 0.05]	1.03	[1.00, 1.06]	0.032

Supplementary Table S8: Results of the best model to predict the probability of an attack in the PSG. The table exhibited results of a logistic regression model using the attack in the PSG as a response variable (0 = do not attack, 1 = attack). The paranoia score and the post-experimental questionnaire items were added as predictors. Each of the five post-experimental questionnaire items was used as a continuous variable. All continuous variables were standardized. The odds ratios were also reported for each predictor. The results reported in the table were generated using model averaging (see Supplementary Method for details). CI: confidence intervals.

Parameters	Estimates	[95% CI]	Odds ratio	[95% CI]	*P* Value
Intercept	−1.96	[−2.38, −1.52]			<0.001
Self-interest	−0.57	[−1.08, −0.06]	0.56	[0.34, 0.94]	0.028
Harmful intent	0.09	[−0.30, 0.48]	1.09	[0.74, 1.61]	0.660
Competitive intent	1.15	[0.59, 1.70]	3.14	[1.81, 5.47]	<0.001
Prediction of fear	−0.18	[−0.65, 0.30]	0.84	[0.52, 1.35]	0.465
Aggressiveness	0.67	[0.31, 1.04]	1.96	[1.36, 2.83]	<0.001
Paranoia	0.02	[−0.15, 0.18]	1.02	[0.86, 1.19]	0.855
Age	0.27	[−0.12, 0.67]	1.31	[0.88, 1.94]	0.178

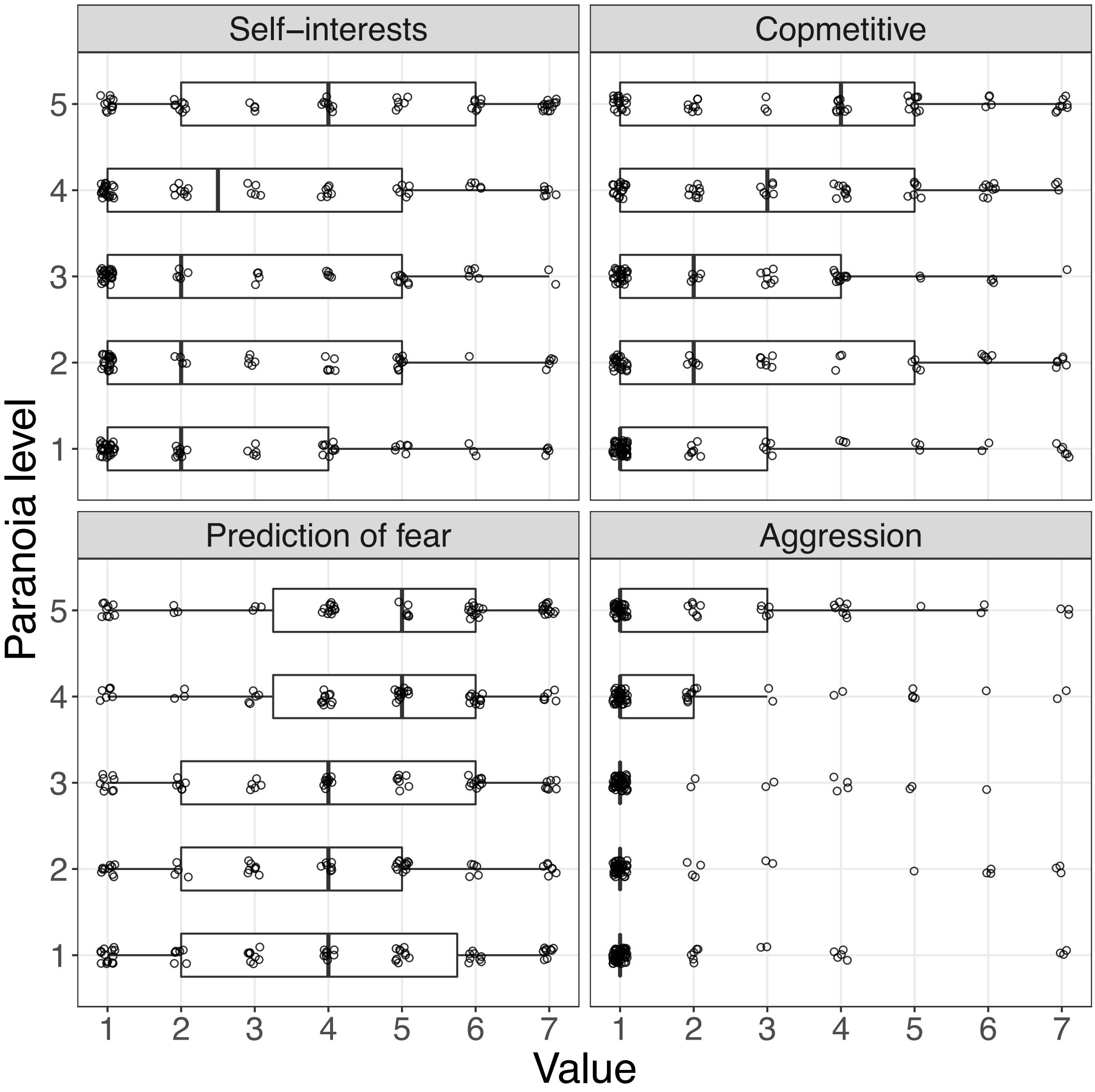

Supplementary Figure S1: Distributions of post-experimental questionnaire items by paranoia levels. The paranoia scores were classified into 5-levels according to quantiles for visualization (1, 32–34; 2, 35–39; 3, 40–50; 4, 51–72; 5, 73–160). Each point represents each participant. Boxplots indicate the distributions of the paranoia score. The box, the thick line in each box, and the whisker represent the interquartile range (IQR), the median, and the distances 1.5 × IQR, respectively. Random jitter was added to each point for ease of visibility.

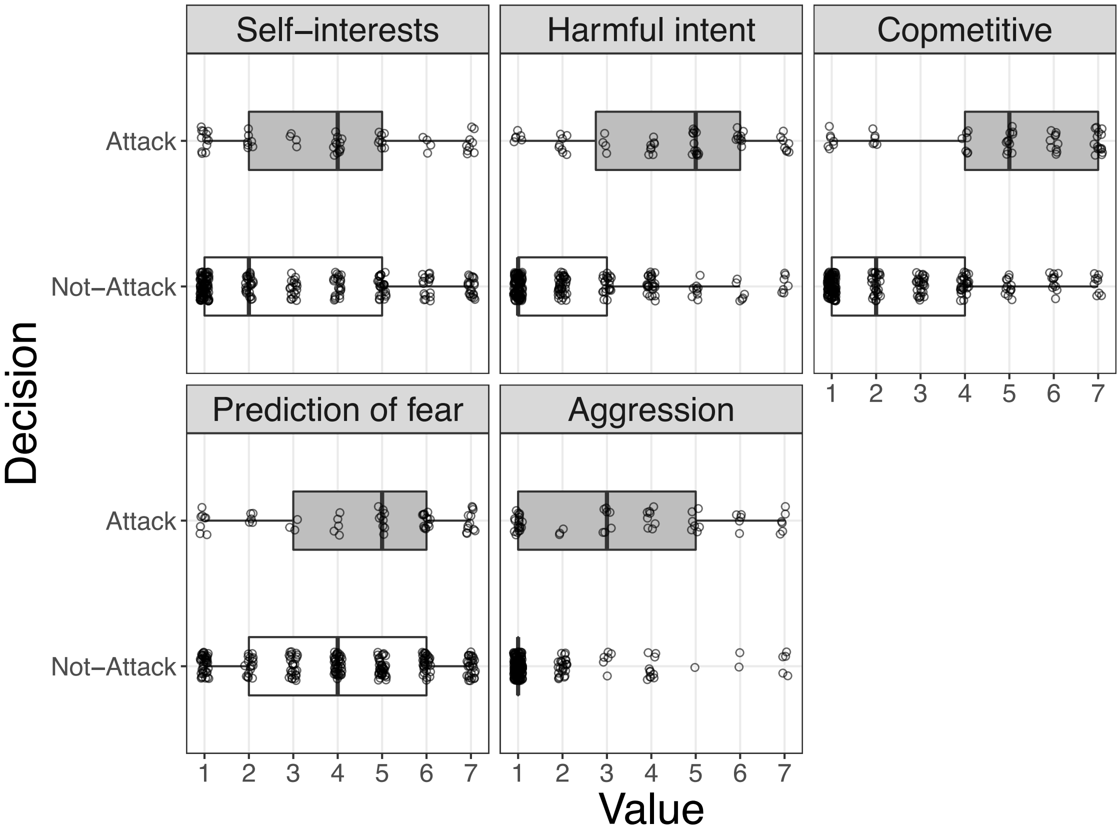

Supplementary Figure S2: Distributions of each score of post-experimental questionnaire items in relation to decisions in the PSG. Each point represents each participant. Boxplots indicate the distributions of the paranoia score. The box, the thick line in each box, and the whisker represent the IQR, the median, and the distances 1.5 × IQR, respectively. Random jitter was added to each point for ease of visibility. The distribution of harmful intent supposition by decisions, which is the same as in Figure 2, is shown for comparison.

Supplementary Method

Model selection with model averaging

The steps for the model selection with multi-model averaging are as follows: 1) We specified a global model comprising paranoia score, five items asked in the post-experiment questionnaire (i.e., the supposition of harmful intent, self-interest, competitive intent, and prediction of the opponent's fear and participant's aggressiveness), and gender and age were incorporated as predictors. 2) The best model was selected from the global model according to the Akaike Information Criterion corrected for small sample sizes (AICc) of each model. The lower the AICc value of the model, the better the model fit to data. The best model, with the smallest AICc, was derived from the global model by comparing all possible combinations of predictors. 3) We selected a "top-level model set," containing models whose AICc values were within two units of the best model. 4) Parameter values of each predictor were obtained by averaging across the top-level models.

Each model in the top-level model set has similar predictive accuracy to the data. The multi-model averaging helps prevent over-reliance on a single best model in estimating parameter values while considering uncertainty about estimations. The multi-model averaging was implemented using the "MuMIn" package for R (Bartoń, 2020). Table 3 reported the full model-averaged coefficients, which were considered as more conservative results.

Bartoń, K. (2020). MuMIn: Multi-Model Inference. R package version 1.43.17. https://CRAN.R-project.org/package=MuMIn

Supplementary Materials

Instructions for the pre-survey to assess the Green et al. (2008) Paranoid Thoughts Scale.

Screen 1
Consent Form

The following is an informed consent form. You are required to read it thoroughly and mark your consent if you agree with the statements. Please note that if you have given your consent, you are still allowed to cancel your participation at any time during the study.

Purpose of the study
The purpose of this study is to investigate people's attitudes and actions/behaviors. Completing this HIT will qualify you for a subsequent HIT at a later date. You will be notified when the subsequent HIT becomes available.

Research Ethics Review Board
The present study has obtained approval by the Research Ethics Review Board at the Department of Psychology, Faculty of Liberal Arts, Teikyo University, Japan.

Time required
The task in this study takes about 5 minutes to complete.

Handling of personal information
Data obtained in the study will be anonymized and processed. Individual participants will not be identified in the study. Even if the data obtained through the study are reported in scientific papers and other media, no data allowing individual participant identification will be published.

Please tick below if you agree to participate in the study. If you would not like to participate, or if you change your mind during the study, please close this window.

☐I have understood the statements mentioned above, and I agree to take part in the study.

Screen 2

You are invited to participate in a simple survey. In this survey, we will ask you to answer questions regarding your attitude.

To begin, please enter your Amazon Mechanical Turk Worker ID here.

Your Worker ID can be found on your dashboard page.

Your Worker ID starts with the letter A and has 12-14 letters or numbers. It is NOT your email address. If we do not have your correct Worker ID, we will not be able to pay you.

Screen 3

Please read each of the following statements carefully. They refer to thoughts and feelings you may have had about others **over the last month**. Think about the last month and indicate the extent of these feelings from **1 (Not at all) to 5 (Totally)**.
Please complete both Part A and Part B.
(N.B. Please do not rate items according to any experiences you may have had under the influence of drugs.)

Part A
1. I spent time thinking about friends gossiping about me.
2. I often heard people referring to me.
3. I have been upset by friends and colleagues judging me critically.
4. People definitely laughed at me behind my back.
5. I have been thinking a lot about people avoiding me.
6. People have been dropping hints for me.
7. I believed that certain people were not what they seemed.
8. People talking about me behind my back upset me.
9. I was convinced that people were singling me out.
10. I was certain that people have followed me.
11. Certain people were hostile towards me personally.
12. People have been checking up on me.
13. I was stressed out by people watching me.
14. I was frustrated by people laughing at me.
15. I was worried by people's undue interest in me.
16. It was hard to stop thinking about people talking about me behind my back.

Screen 4

Please read each of the following statements carefully. They refer to thoughts and feelings you may have had about others **over the last month**. Think about the last month and indicate the extent of these feelings from **1 (Not at all) to 5 (Totally)**.
Please complete both Part A and Part B.
(N.B. Please do not rate items according to any experiences you may have had under the influence of drugs.)

Part B
1. Certain individuals have had it in for me.
2. I have definitely been persecuted.
3. People have intended me harm.
4. People wanted me to feel threatened, so they stared at me.
5. I was certain people did things in order to annoy me.
6. I was convinced there was a conspiracy against me.
7. I was sure someone wanted to hurt me.
8. I was distressed by people wanting to harm me in some way.
9. I was preoccupied with thoughts of people trying to upset me deliberately.
10. I couldn't stop thinking about people wanting to confuse me.

11. I was distressed by being persecuted.
12. I was annoyed because others wanted to deliberately upset me.
13. The thought that people were persecuting me played on my mind.
14. It was difficult to stop thinking about people wanting to make me feel bad.
15. People have been hostile towards me on purpose.
16. I was angry that someone wanted to hurt me.

Screen 5

What is your gender?

〇Male
〇Female

What is your age?
____

Screen 6

Thank you very much for taking part in our study.

The Secret Key will be displayed on the next page.
Please make sure to enter your Secret Key in your HIT window before submitting your HIT.

After all the data for this survey has been collected, you will receive an invitation to the next HIT by email at a later date.

Instructions for the preemptive strike game

Screen 1

Consent form

The following is an informed consent form. You are required to read it thoroughly and mark your consent if you agree with the statements. Please note that if you have given your consent, you are still allowed to cancel your participation at any time during the study.

Research Ethics Review Board
The present study has obtained approval from the Research Ethics Review Board at the Department of Psychology, Faculty of Liberal Arts, Teikyo University, Japan.

Purpose of the study
The purpose of this study is to investigate people's attitudes and actions/behaviors.

Time required
The task in the study takes about 5-10 minutes to complete.

Handling of personal information
Data obtained in the study will be anonymized and processed. Individual participants will not be identified in the study. Even if the data obtained through the study are reported in scientific papers and other media, no data allowing individual participant identification will be published.

Please tick below if you agree to participate in the study. If you do not wish to participate, or if you change your mind during the study, please close this window.

☐I have understood the statements mentioned above, and I agree to take part in the study.

Screen 2

To begin, please enter your Amazon Mechanical Turk Worker ID here.

Your Worker ID can be found on your dashboard page.

Your Worker ID starts with the letter A and has 12-14 letters or numbers. It is NOT your email address. If we do not have your correct Worker ID, we will not be able to pay you.

Screen 3

Introduction
Individuals participating in the study will receive $0.50 as remuneration. They will also receive a bonus according to the results of this experiment.

Instruction

In this experiment, you will be paired with another participant and will **decide whether to press the button displayed on your computer screens**.

- First, you and your partner will each receive $0.50 as capital.
- If neither you nor your partner press the button for 30 seconds, both you and your partner will receive the $0.50 as is.
- If either you or your partner presses the button first, the person pressing it first will lose $0.10 from their capital, and the amount acquired will fall to $0.40. However, the person whose partner presses the button first will lose $0.50 from their capital, so they will end up with nothing.

This experiment will be carried out **just once**.

The amount earned in this experiment will be paid as a bonus.

Each participant will make a decision separately. After we finish collecting all data, we will randomly make pairs of participants, and your bonus will be determined by this random pairing process.

To confirm whether you have understood the rules of the experiment or not, we will ask you some questions.

1. If neither you nor your partner pressed the button for 30 seconds, how much money will each of you get?

   You: $0, $0.40, or $0.50 (the correct answer is $0.50)
   Your partner: $0, $0.40, or $0.50 (the correct answer is $0.50)

2. If **you** pressed the button before your partner did, how much money will each of you get?

   You: $0, $0.40, or $0.50 (the correct answer is $0.40)
   Your partner: $0, $0.40, or $0.50 (the correct answer is $0)

3. If **your partner** pressed the button before you did, how much money will each of you get?

   You: $0, $0.40, or $0.50 (the correct answer is $0)
   Your partner: $0, $0.40, or $0.50 (the correct answer is $0.40)

Once you complete these questions, please click 'Next.' If you answer all questions correctly, you can proceed to the Decision Screen.

Please note that you cannot proceed to the following pages and receive your bonus if you do not answer all questions correctly.

<u>Screen 4</u>

We will begin shortly. Please wait. Do not touch the computer.

<u>Screen 5</u>

The Decision Screen

Time left to complete this page: **0:30**

Please decide whether to press the button or not.

Button

<u>Screen 6</u>

Questions

While you were deciding whether to press the button or not, to what extent did you think about the following? Please read the sentences below, and choose the number that best applies to your thoughts (1: I did not think so at all - 7: I thought so strongly).

"My partner must be planning to earn lots of money."
○1: Not at all ○2 ○3 ○4 ○5 ○6 ○7: Strongly

"My partner must be planning to reduce the money I get."
○1: Not at all ○2 ○3 ○4 ○5 ○6 ○7: Strongly

"My partner must be planning to get more money than me."
○1: Not at all ○2 ○3 ○4 ○5 ○6 ○7: Strongly

"My partner must be afraid that I will press the button first."
○1: Not at all ○2 ○3 ○4 ○5 ○6 ○7: Strongly

"I want to reduce the amount of money my partner gets."
○1: Not at all ○2 ○3 ○4 ○5 ○6 ○7: Strongly

Once you complete these questions, please proceed to the next page.

Screen 7

This concludes the experiment.

Once all the experiments are completed, we will compute bonuses. The bonuses will be paid within 10 days of completing all the experiments. To receive your bonus, please make sure to enter the Secret Key shown below in your HIT window before submitting your HIT.

Your Secret Key is:
XXXXXXXXXXX

You may close this page after having confirmed your Secret Key. Thank you very much for taking part in our study.
